# Evolutionary Divergence in Brain Size between Migratory and Resident Birds

**DOI:** 10.1371/journal.pone.0009617

**Published:** 2010-03-10

**Authors:** Daniel Sol, Núria Garcia, Andrew Iwaniuk, Katie Davis, Andrew Meade, W. Alice Boyle, Tamás Székely

**Affiliations:** 1 Centre for Ecological Research and Applied Forestries (CREAF), Autonomous University of Barcelona, Bellaterra, Catalonia, Spain; 2 CEAB-CSIC (Centre for Advanced Studies of Blanes-Spanish National Research Council-Spanish National Research Council), Blanes, Catalonia, Spain; 3 Canadian Centre for Behavioural Neuroscience, Department of Neuroscience, University of Lethbridge, Lethbridge, Alberta, Canada; 4 School of Biological Sciences, University of Reading, Reading, United Kingdom; 5 Division of Ecology and Evolutionary Biology, University of Glasgow, Glasgow, United Kingdom; 6 Department of Biology, University of Western Ontario, London, Ontario, Canada; 7 Department of Biology and Biochemistry, University of Bath, Bath, United Kingdom; Raymond M. Alf Museum of Paleontology, United States of America

## Abstract

Despite important recent progress in our understanding of brain evolution, controversy remains regarding the evolutionary forces that have driven its enormous diversification in size. Here, we report that in passerine birds, migratory species tend to have brains that are substantially smaller (relative to body size) than those of resident species, confirming and generalizing previous studies. Phylogenetic reconstructions based on Bayesian Markov chain methods suggest an evolutionary scenario in which some large brained tropical passerines that invaded more seasonal regions evolved migratory behavior and migration itself selected for smaller brain size. Selection for smaller brains in migratory birds may arise from the energetic and developmental costs associated with a highly mobile life cycle, a possibility that is supported by a path analysis. Nevertheless, an important fraction (over 68%) of the correlation between brain mass and migratory distance comes from a direct effect of migration on brain size, perhaps reflecting costs associated with cognitive functions that have become less necessary in migratory species. Overall, our results highlight the importance of retrospective analyses in identifying selective pressures that have shaped brain evolution, and indicate that when it comes to the brain, larger is not always better.

## Introduction

Understanding the factors influencing the changes in brain size has been an area of great interest to evolutionary biologists since Darwin [1], who believed that the large size of the human brain was closely associated with its higher cognitive capacities. After more than a century of research, however, controversy remains regarding the selective pressures that have driven the enormous diversification in brain size. One reason is that previous studies have mostly focused on documenting advantages and/or costs of the brain under present ecological conditions [reviewed in 2], [3], [4]. These studies have yielded a number of important discoveries such as that larger brains are associated with enhanced ecological opportunism [5], [6], stronger social relationships [7], occupation of more variable climates [8], higher survival in novel environments [9], [10], and less pronounced population decline when the habitat changes [11]. In the absence of historical evidence, however, these findings are by themselves insufficient to understand the evolutionary pressures that have favored the diversification in brain size. This is because the observation that a certain variable is associated with differences in brain size does not necessarily imply that this is the cause of such differences; rather, it may be a consequence [12]. Even when the causal link can reasonably be inferred, there is no guarantee that the evolutionary processes currently operating are the same that operated in the past [13]. The corollary is that to fully understand brain evolution it is critical to adopt a retrospective approach that allows reconstructing the order and direction of the past evolutionary events that led to current patterns [14], [15]. Unfortunately, the rarity of studies using this approach has frustrated efforts to understand the selective pressures that have driven current differences in brain size. In this study, we combine prospective and retrospective phylogenetic-based comparative approaches to assess whether and how brain size has diverged among passerine birds differing in their adaptive response to seasonal environments.

When facing seasonal changes in the environment, birds display two distinct strategies: some birds migrate to less severe regions for the harshest season whereas others remain in the same region throughout the whole year. Previous work has shown that these distinct strategies are associated with differences in some brain structures. Migratory dark-eyed juncos (*Junco hyemalis*), for example, have a higher density of neurons in the hippocampus than resident juncos [16]. Differences are not, however, restricted to small, specialized regions of the brain. Analyses of passerine birds from the Palearctic region suggest that the size of the whole brain, relative to body size, is significantly smaller in migratory species than it is in resident ones [17], [18], [see also 19]. The reasons for these differences in overall brain size remain obscure, although various hypotheses have been proposed. First, natural selection could have favored larger brains in resident species if this enhances their behavioral flexibility to face sharp seasonal changes in resources [18]. This hypothesis is based on the observation that changes in food availability among seasons is a major cause of migration [20], [21], [22] and that larger brains (relative to body size) are associated with increased behavioral flexibility to explore and utilize new or changing food resources more successfully [5], [6], [9], [10], [11], [23]. Second, selection could have favored a decrease in the brain of migratory species due to costs associated with migration [17], [18], [24]. Growing a large brain requires a long developmental period [25] and is energetically demanding [26], which may be excessively costly for migratory birds that have to travel long distances and that have a short time period available for reproduction. Third, selection may have favored migratory behaviors if a relatively small brain has decreased the ability of individuals to cope with the difficulties of harsh winters, forcing them to move to more favorable regions. If so, the observed differences in brain size between migratory and resident species would not be the consequence, but rather the cause of differences in migratory strategy [18]. Finally, the differences in overall brain size between migratory and resident species could be a spurious result caused by confounding factors and/or systematic errors in the brain size measures [27].

The aims of our study are therefore to determine both whether and how the brain has diverged between migratory and resident birds. To address these issues, we compiled information on brain volume and migratory distance from published studies and our own work for 600 passerine species ranging from arctic to tropical regions. Because we found that the brain-migration association is highly significant and cannot be explained on the basis of measurement errors, phylogenetic effects or other potential confounding factors, we next used a retrospective phylogenetic-based approach to examine whether brain size and migration are tightly coupled over evolutionary time, appearing and being lost simultaneously, or if rather, changes in one trait have facilitated changes in the other. Reconstructing evolutionary transitions in a phylogeny of contemporary species is not easy, but in some cases the order and direction of the evolutionary changes can be inferred with phylogenetic Markov chain methods [28], [29], [30], [31]. We used such a phylogenetic framework in an attempt to clarify which of the hypothesized evolutionary scenarios is more likely to account for the brain-migration association. Finally, we further explored the best supported scenarios with path analyses, which allowed us to assess to what extent the brain-migration association was caused by direct effects or by indirect effects associated with environmental, energetic and/or developmental factors.

## Results and Discussion

**Figure 1:**
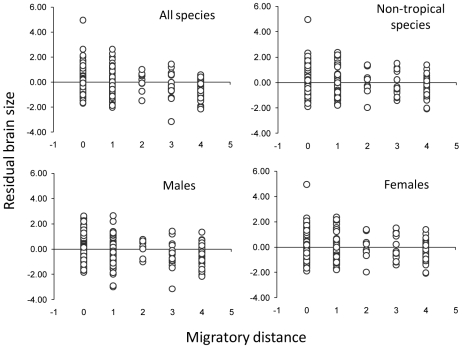
Relationship between brain residual size and migratory distance in passerine birds. A positive brain residual indicates that the species has a brain larger than expected by their body size whereas a negative brain residual indicates that the brain is smaller. Migration has been coded as follows: 0) no populations of species is migratory anywhere in its distribution, 1) altitudinal movements and other movements less than 100 km, 2) movements between 100 and 700 km, 3) 700–1500 km, and 4)>1500 km.

Brain volume is negatively associated with migratory distance, when the allometric effect of body mass is controlled for (PGLS using single-source brain data: *t* = −5.47, *P*<0.0001, Partial *R^2^* = 0.07, λ = 0.89, *N* = 351 species, Fig. 1). This pattern holds when excluding species with fewer than three specimens measured (*t* = −4.08, *P*<0.0001, Partial *R^2^* = 0.09, λ = 0.95, *N* = 151 species), and when the analysis is run separately for males (*t* = −4.69, *P*<0.0001, Partial *R^2^* = 0.06, λ = 0.68, *N* = 313 species) and females (*t* = −4.67, *P*<0.0001, Partial *R^2^* = 0.07, λ = 0.58, *N* = 285 species; Fig. 1). To investigate the generality of these results, we repeated the analysis with the full dataset of 600 species that combined brain information from different sources. This analysis confirms that small-brained passerines tend to migrate longer distances than large-brained species (PGLS multiple-source brain data: *t* = −6.96, *P*<0.0001, Partial *R^2^* = 0.07, λ = 0.78), thus generalizing and extending previous results [17], [18], [19].

Given the correlative nature of the evidence provided, there is a risk that the reported association is spuriously caused by the effect of confounding factors. A number of factors could potentially have confounded the brain-migration relationship, including the degree of seasonality in the environment, the extent to which the habitat buffers individuals from climatic conditions, the temporal fluctuations in resource availability and intrinsic features of the species related to energetic demands, developmental periods and social behavior [2]. However, the brain-migration association cannot be explained on the basis of any of these factors (Table 1). Overall, our results provide the clearest and most general support to date for genuine differences in relative brain volume between migratory and resident species.

**Table 1:** Brain mass as a function of migratory distance, with extrinsic and intrinsic factors susceptible to influence the relationship incorporated as covariates.

Variable	Parameter	SE	*t*	*P*
Migratory distance	−0.025	0.006	−4.17	<0.0001
Distance to equator	0.001	0.001	0.07	0.9476
Insectivorous diet	−0.051	0.031	−1.58	0.1198
Frugivorous diet	−0.072	0.055	−1.32	0.1912
Occurrence in forests	0.019	0.018	1.11	0.2705
Incubation period	−0.016	0.008	−1.97	0.0532
Fledging period	0.004	0.003	1.59	0.1177
BMR	0.026	0.080	0.32	0.7463
Social monogamy	−0.038	0.026	−1.48	0.1444
Body mass	0.633	0.054	11.63	<0.0001

The model is based on 74 species for which information on all variables was available. The coefficients are the slopes (continuous variables) or mean differences (binary variables) of the relationship between log-brain mass (response variable) and all the variables (predictors) estimated with the method of the phylogenetic generalized least squares and the phylogeny branch lengths set to one [57].

**Figure 2:**
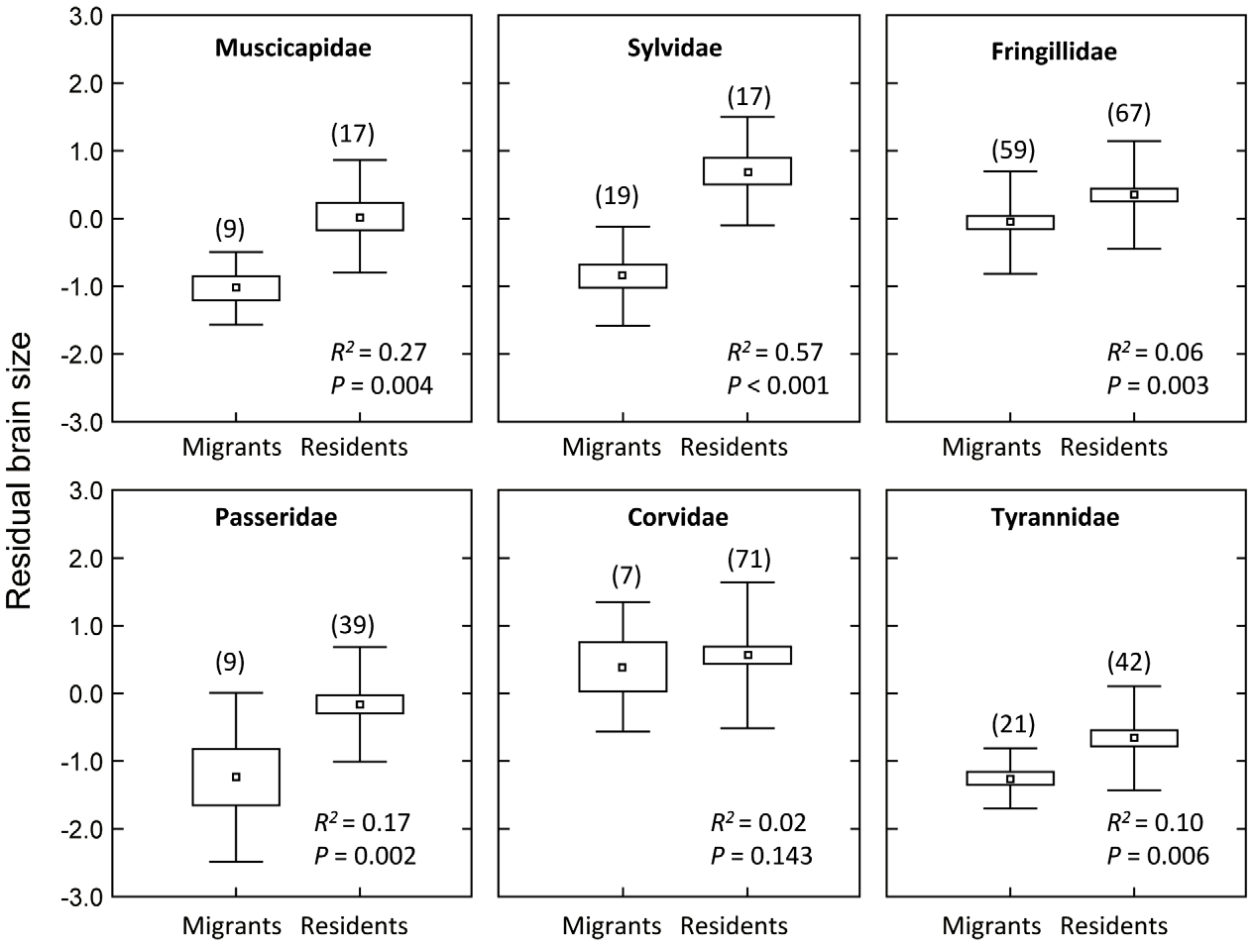
Differences in residual brain size between resident migratory species within families. The six families correspond to those for which enough species were available for the analyses. Differences are expressed as mean ± SE ± SD. Residence includes migratory distances 0 and 1 (see methods) and migration distances 2, 3 and 4. The *R^2^* and *P* values come from a PGLS in which residual brain size was the response variable and migratory behavior the predictor.

The limited explanatory power of the brain-migration association is not surprising considering the variety of environmental factors that may influence brain size and migratory behavior. It should also be noted that migratory distance is difficult to quantify, and thus estimates are subject to error, which might detract from our ability to resolve the actual strength of its association with brain size. However, it is noteworthy that with large datasets it is possible to detect very small effects, the biological relevance of which is disputable [32]. To better grasp the strength of the brain-migration association, we compared brain size of migratory and resident species from the same taxonomic family, assuming that closely-related species are more likely to have been subject to similar selective pressures. This analysis confirms the existence of significant differences in brain size between migratory and resident species in five out of the six families examined, and suggests that the divergence has been more important in some families than in others (Fig. 2). Thus, while in the Sylvidae, Muscicapidae and Passeridae migratory behavior explains a substantial fraction of the variation in residual brain size (range 0.17–0.57, Fig. 2), in the Tyrannidae and Fringillidae the fraction explained is significant yet low (0.10 and 0.06, respectively), and in the Corvidae the fraction is not statistically significant. We can only speculate on the reasons for such differences, but one obvious possibility is that the selective forces that shaped the evolutionary divergence in brain size differ among families [2], [33], [34].

**Figure 3:**
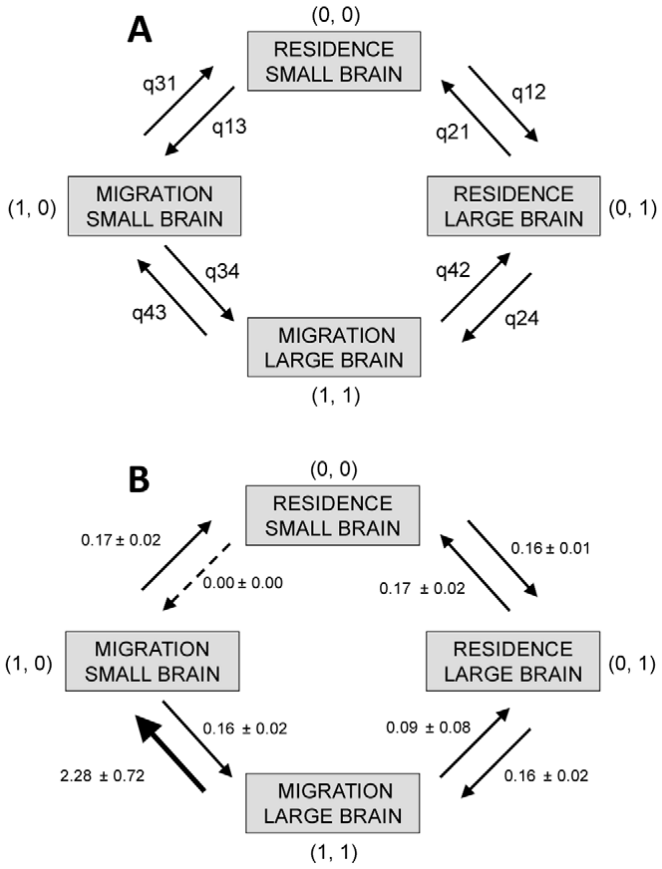
Directional evolution of migratory behavior and brain size in passerines. (A) general model with parameter notations and (B) model with parameters estimated based on 377 non-tropical breeding species. Each *q_i_* represents the likelihood and associated standard deviation of a transition, estimated with Bayesian approaches based on a phylogeny with branch length set to 1.

Having shown that the brain-migration association is robust, we then may ask how the association has evolved. To address this question, we adopted an historical perspective. We started by reconstructing the probable ancestral states for both traits following the Bayesian MCMC method described by Pagel et al. [35]. We found a posterior probability of 90.7%±3.2% for a large brain and of 87.5%±5.5% for residence being the ancestral states of the passerines. Next, we used the MCMC approach [31] to detect the order and direction of evolutionary changes in the brain-migration association (Fig. 3A). The log-Bayes Factor ranges from 3.04 to 18.31, depending on the phylogeny used, further supporting the model of correlated evolution. The most visited models suggest that parameter *q_13_* is zero (98.97% of time), and that all the other parameters except *q_43_* are in the same rate category (Fig. 3B). The reason why models of correlated evolution predominates in the posterior sample is explained by the fact that *q_43_*>*q_21_* (99% of time) and *q_24_*>*q_13_* (98.8% of time). Thus, although our results do not deny the case-by-case importance of the three proposed evolutionary scenarios, the most likely evolutionary pathway is that migratory behavior changed first in large-brained lineages and that migration selected for smaller brains. In agreement with the scenario, *q*_43_ is greater than *q*_34_ more than 99.9% of the time, indicating that in migratory lineages the brain was more likely to decrease than to increase. The alternative route in which brain size changes first and this forces small-brained species to migrate is not supported because *q_13_*≈0.

**Figure 4:**
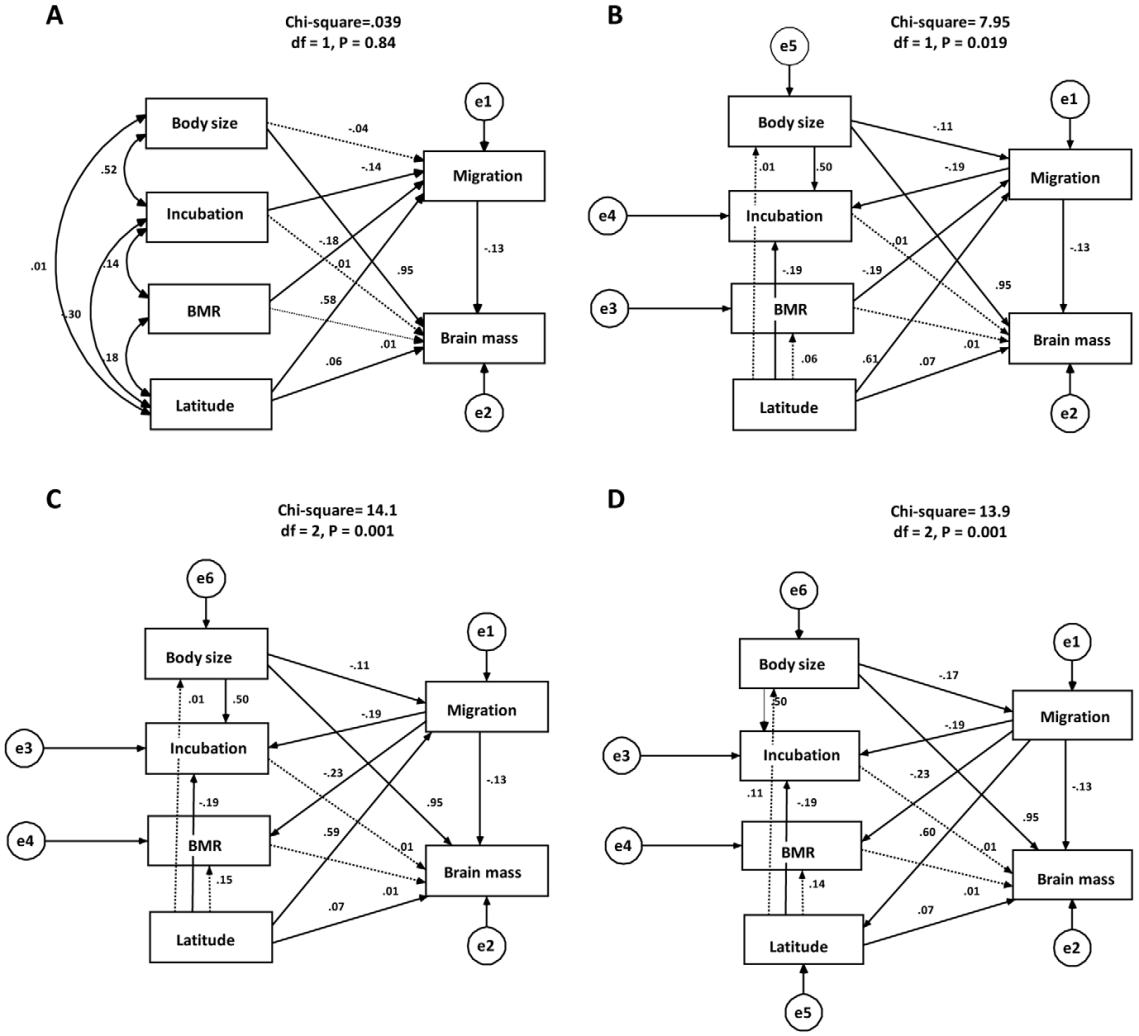
Four best path models (A–D) deconstructing direct, indirect and spurious effects in the relationship between brain mass and migratory distance. The path coefficients and fit of the models were calculated based on the correlation matrix of raw values from all species (600 species), with means and intercepts estimated to deal with missing values [63]. Solid lines indicate the paths that are significant at P<0.05. All path coefficients that are significant are also significant when tested with the phylogenetic generalized least squares approach. Brain mass (Brain), body mass (Body) and incubation period (Incubation) were log transformed to improve normality. Although migration distance (Migration) is measured in an ordinal scale (see methods), the residuals from the model in which this variable is used as response variable fit well to a normal distribution. The terms e1–e6 refer to the error terms.

Why should a migratory life style favor smaller brains? As discussed above, migratory behavior is thought to impose important energetic and developmental costs. The relevance of these costs cannot be demonstrated with a comparative approach, but we can at least try to evaluate the validity of a set of plausible scenarios with path analyses. From all the models we tested, only one provides a good fit to the data (Fig. 4A). This model suggests that the brain-migration association is in part caused by unanalyzed effects [sensu 36] associated with the correlation between BMR, incubation period and body mass, once latitude is taken into account. However, the path model also indicates that an important fraction (over 68%) of the correlation between brain mass and migratory distance came from a direct effect of migration on brain size. This direct effect could reflect limitations in the variables used for the analyses. For example, if affording a large brain is compensated by a decrease in other metabolically expensive organs [37], then it is unlikely that correlations between current patterns of BMR and brain size may be observed. Alternatively, the direct effect of migration on brain size could reflect costs associated with cognitive functions that have become less important in migratory species. We suggest that one of the brain areas meriting particular study in this context are the pallial areas of the telencephalon, which are thought to be involved in the executive functions that allow learning and behavioral innovation [5], [38]. These brain structures make up a substantial portion of the brain, implying that the sizes of these higher processing centers can essentially be predicted from overall brain size [38], [39]. A reduction of these areas, and hence of the whole brain, is expected if, as suggested by Mettke-Hoffmann and Greenberg [40], in migratory species the information gathered by individuals as they travel through novel environments is only useful for short periods and information relevant to one environment may also expose individuals to risks (e.g. novel predators) in another. The implication is that learning and innovation may be more costly than beneficial in migratory species, which should favor “innate” behaviors over flexible behaviors [see also 41]. Although still limited, there is some evidence that migratory passerines are less exploratory [42] and have a lower propensity for feeding innovations [18] when compared to resident species.

The current paradigm in brain evolution research primarily focuses on how species increase overall brain size, assuming that larger is always better [43]. In this paradigm, the importance of selection for smaller brains is under-appreciated, perhaps because researchers in brain evolution are primarily interested in highly encephalized animals. However, if the costs of producing and maintaining the brain outweigh the benefits, then selection should favor a decrease in brain size [15], [26], [43]. Our analyses suggest that it is possible to identify such evolutionary episodes provided that we move beyond the classical prospective approach and start using phylogenetic-based approaches that highlight how present-day diversification in brain size can be understood as a result of historical events.

## Materials and Methods

### Species Information

Passerine birds are ideal for our study because they show substantial variation in both brain size and migratory behavior [18], [44]. Our analyses were based on species from all geographic regions for which information on brain size, migratory strategy and phylogenetic relationships were available (Text S1, Table S1).

### Brain Size Estimations

We focused on the relative size of the whole brain instead of the size of brain components, mainly due to data availability. Nevertheless, we think that analysis of whole-brain is justified in our case for three main reasons. First, many brain component volumes are tightly correlated with whole-brain volumes, particularly the large parts of the brain such as the avian pallial areas that are associated with innovation and learning [3]. Second, many regions distributed throughout the brain are activated in learning and decision making processes [3]. Third, if the association between brain size and migration reflects energetic and/or developmental costs, rather than cognitive demands, then these costs should be easier to detect if we examine the whole brain than if we focus on small brain areas. In any case, whether differences between migratory and resident species are related to the whole-brain or component volumes is an empirical issue that should be addressed when appropriate data become available.

A major concern of comparative studies of brain size is that different authors often use different measurement methods [27], which may introduce biases in the measures and lead to spurious relationships, particularly when sample sizes are small [45], [but see 46]. To tackle this problem, we initially estimated brain volumes using a single technique, the endocranial volume technique, which calculates brain volumes by filling the skulls of museum specimens with lead shot [45]. Although this is an indirect measure of brain size, the advantage is that the endocranial cavity does not change with age as long as skull development is complete. We consequently only used data from adults, identified based on plumage and/or skull pneumatization. Endocranial measurements are also not biased by histological techniques (shrinking, freezing, desiccation) that can cause variation in the measurement of fresh brains. The skull endocasts for 4,053 specimens from 351 species were measured by a single investigator (Andrew Iwaniuk). These measures of brain volume were highly repeatable within species, with 98.7% of variation found among species rather than within species. The sex of the specimens (male or female) and its body mass were also recorded whenever this information was available. The brain volume of some rare species was estimated based on few specimens (i.e., <3), which may affect the accuracy of the measures. However, the conclusions hold when these species were excluded from the analyses (see results). When information on brain volume was available from two or more specimens, we estimated brain size by using the average.

Endocranial volumes can be converted to mass by multiplying the reported value by the density of fresh brain tissue [1.036 g ml–1, 45]). This allowed us to combine our endocranial volumes with brain masses published in the literature. Despite the concerns raised about the problems of combining brain measures from different methods, there was a strong correlation between brains estimated by the endocranial technique and those estimated by weight (*r_69_* = 0.985, *P*<0.0001), consistent with previous analyses [45]. Therefore we conducted a second set of analyses with the combined brain measures, thereby increasing sample size from 351 to 600 species.

Previous work in birds has shown that it is not brain size per se, but the extent to which the brain is either larger or smaller than that expected for a given body size which indicates adaptation for enhanced neural processing [5], [6]. To remove the allometric relationship with body size, we modeled the association between absolute brain size (response variable, log-transformed) as a function of migratory distance (predictor) while including body mass (log-transformed) as covariate in the model. In addition, we estimated the residuals of a log–log least-square linear regression of brain mass against body mass (residual brain size, hereafter), and modeled these residuals as a function of migratory distance. This second approach is equivalent to the first, and yielded similar results, and we only used it to estimate the proportion of variation in brain size relative to body size that was explained by migratory distance (Partial *R^2^*, hereafter). The residuals were also used to build the graphics. The body mass of species was taken either from the same specimens for which we measured the brain volume or from published sources, when such information was unavailable. For analyses within sexes we used sex-specific body masses.

### Migration Data

Quantitative data on migratory behavior are scarce, especially from tropical regions, so to expand the species coverage we followed Boyle and Conway [47] and scored migratory behavior on an ordinal scale: 0) no populations of species is migratory anywhere in its distribution, 1) altitudinal movements and other movements less than 100 km, 2) movements between 100 and 700 km, 3) 700–1500 km, and 4)>1500 km. By classifying a species as resident, we do not imply that it lacks any pre-adaptation for migration, but simply that it does not currently display migratory movements. We assigned a species to the shortest migratory distance category (1) when at least some populations of that species were known to migrate locally. For northern hemisphere migrants, we measured the shortest distance between the reported northern edge of the non-breeding range and the northern edge of the breeding range, whereas for south-hemisphere migrants we measured the shortest distance between the reported southern edge of the non-breeding range and the southern edge of the breeding range. For partially migratory species, we used the longest estimate of migratory distance.

### Environmental Variables

Migratory behavior can be affected by the degree of seasonality in the environment where the species occurs [47], [48]. We assembled published information (see Text S1) on environmental variables that reflect the seasonality in the environment: (i) latitude, (ii) occurrence in buffered breeding habitats; and (iii) use of temporally variable food items. The first of these, latitude, was measured as maximum latitudinal degrees of the breeding range from equator. The use of buffered breeding habitats [49] was quantified as whether the species occurs either in forests (i.e. the habitats that offer more protection from climatic fluctuations) or in more open habitats (i.e. less climatic buffered habitats). Finally, the use of temporally variable diet types was quantified in two variables as whether the species' diet is primarily based on insects (a resource that changes seasonally in temperate and polar regions; see Newton [50]) and/or fruits (a resource that requires high mobility to be tracked; see [48] and [47]).

### Intrinsic Features of Species

The costs of growing and maintaining a large brain in migratory birds may emerge from limited time for development and the energetic costs of travelling long distances [17]. Social behavior may, in turn, favor residence in large brained birds by facilitating a more efficient exploitation of seasonal resources [47]. We used the following proxy variables to represent these features: (i) development period, measured as the number of days of incubation (incubation period) and the days from hatching to fledging (fledging, see [25]; (ii) basal metabolic rate [26], in kilocalories per day; (iii) body mass (see above); and (iv) social pairbonding, defined as whether the species is monogamous or it is not [2].

### Phylogenetic Hypothesis

Our passerine phylogeny was extracted from the avian supertree developed by Katie Davis and Rod Page (University of Glasgow). The source data for the avian supertree were collected and processed following Bininda-Emonds et al. [51]. The supertree assembles information from 748 published phylogenetic trees. The matrix was run in TNT (Tree analysis using New Technology), developed by P. A. Goloboff, J. S. Farris, and K. C. Nixon [52], which found a single most parsimonious tree of length 17899. The phylogeny and further details are available in Davis ([[53], http://theses.gla.ac.uk/178/]). Following Perez-Barbería et al. [54], we calculated branch lengths using three different methods [55], [56], [57]. Sensitivity analysis demonstrated that the results did not depend upon which branch lengths were used and in the text we report those based on a phylogeny with branch length set to 1.

### Phylogenetic-Based Prospective Comparative Methods

We modeled brain size (both as log-absolute brain size and residual brain size) as a function of migratory distance and confounding factors with a phylogenetic generalized least squares (PGLS) approach [58], [59]. This method is based on the estimation of a parameter λ, which measures the degree to which the variance/covariance matrix follows the Brownian model. We simultaneously estimated λ and fitted GLS models, using the R-package Ape [60] and an R code kindly provided by R. P. Freckleton [58]. Diagnostic plots were examined to check for outliers and heteroscedasticity, as well as to ensure the normality of errors.

### Phylogenetic-Based Retrospective Comparative Analyses

The evolutionary transitions that generated the brain-migration association were evaluated with phylogenetic Markov chain methods, implemented in the DISCRETE option from BAYES-TRAITS [28], [30]. We used both maximum likelihood (ML) and Bayesian Monte Carlo (MCMC) methods to derive point estimates of log-likelihoods and the parameters of statistical models [28], [30], [35]. Because both approaches yielded consistent results, we report the results from the Bayesian MCMC approach. The Bayesian MCMC is more robust because it provides the confidence intervals of the parameters whereas ML only gives the best values.

DISCRETE tests for correlated evolution between two binary traits by comparing the fit of two continuous-time Markov models. One of these is a model in which the two traits evolve independently on the tree. This model is defined with two rate coefficients per trait. The dependent model allows the traits to evolve in a correlated fashion such that the rate of change in one trait depends upon the background state of the other. The dependent model can adopt four states, one for each combination of the two binary traits. In the MCMC approach, these two models are compared with the log-Bayes Factor test (BF), which is: 2log[harmonic mean(dependent model)]–log[harmonic mean(independent model)]. Values of 2–5 on a log scale are “positive” evidence for correlation between the studied traits, greater than 5 is “strong” evidence, and greater than 10 is “very strong” evidence.

As with all Bayesian methods, the results of the MCMC method are qualified in terms of the data, the model and the priors [31]. We used a uniform prior on the models and an exponential prior on the rate coefficients [31]. We ran ten independent Markov chains of each model, all of which converged on to similar likelihood and parameter values. We applied the Reversible-jump (RJ) Discrete Markov chain model [31] to the trait data to integrate the results over all possible combinations of the models. This allowed us to identify those models most frequently visited during the construction of the dependent model. We ran the RJ Discrete Markov chain for at least 101,000,000 iterations, so that the chain had ample opportunity to visit the various models. We discarded the first 1,000,000 iterations of each run, and then we sampled every hundredth iteration, to produce 1,000,000 sampled points. This sampling frequency is adequate to produce effectively independent samples [31]. All 10 runs gave the same results and we report the first one here.

DISCRETE requires the variables studied to be binary. We considered a species as migratory when any source reported migratory movements more than 100 km and resident otherwise. Migratory behavior is generally considered to be an evolutionary labile attribute [61]. Highly evolutionary labile traits retain phylogenetic information for relatively short times and hence can be difficult to reconstruct over evolutionary time. However, our analyses suggest that migratory behavior shows substantial phylogenetic conservatism (λ = 0.797–0.922, depending on the phylogeny used).

An obvious way to convert brain masses into a binary trait is to split species according to whether they have negative or positive brain residuals in a log–log least-squares regression of brain mass against body mass [62]. A negative brain residual indicates that the brain is smaller than expected by the size of the species whereas a positive brain residual indicates that the brain is larger than expected.

One limitation of the Markov chain approach is that many species in the data set breed in tropical regions, where seasonal fluctuations in resources are generally not as pronounced as in temperate/polar regions. Thus, some evolutionary transitions in brain size under residence, particularly the parameter *q_21_*, do not necessarily reflect responses to seasonal environments, but may arise from other pressures. In the PGLS approach, we controlled for this effect by including latitude as a co-variate, but this was not possible in DISCRETE as the method only allows bi-variate comparisons. We tackled this limitation by restricting the analysis to non-tropical breeding species (*N* = 377).

### Path Analysis

We used path analysis to decompose the correlation between brain size and migratory distance as a function of the most relevant extrinsic and intrinsic factors (see results). A path analysis is a multivariate statistical method useful to describe the direct, indirect and spurious dependencies among a set of variables and it is particularly powerful to identify plausible causal scenarios that can then be validated with experiments [36], [63], [64], [65]. We built path analyses using AMOS 16.0 [63], fitting general structural equation models by the method of maximum likelihood with multinormal errors [66]. The path coefficients and fit of the models were estimated based on information from all species (600 species), with means and intercepts estimated to deal with missing values [63]. The fit of the models was evaluated with a chi-square test comparing the observed and predicted covariance matrices [64].

## Supporting Information

Text S1 Sources for data.
(0.03 MB DOC)

Table S1 Migratory distance, body mass, brain mass and source of brain data for the species included in this study. Endocranial volumes were converted to mass by multiplying the reported value by the density of fresh brain tissue [1.036 g ml–1, 1]).
(0.90 MB DOC)
