# Supplementary material for: Evolutionary Divergence in Brain Size between Migratory and Resident Birds
Source: PLoS One. 2010 Mar 10;5(3):e9617. doi: 10.1371/journal.pone.0009617 (PMC2835749; doi:10.1371/journal.pone.0009617)
Supplement: Text S1 — Sources for data. (0.03 MB DOC) [file pone.0009617.s001.doc]

Baker, K. 1997. *Warblers of Europe, Asia and North Africa*. C. Helm, A & C, London.

Boyle, A.W. and Conway, C.J. 2007. Why migrate? A test of the evolutionary precursor hypothesis. Am. Nat., **169**: 344–359.

Cheke, R.A., Mann, C.F. and Allen, R. 2001. *Sunbirds.* C. Helm, A & C, London

Clement, P. 2000. *Thrushes.* Princeton University Press, Princeton.

Clement, P., Harris, A. and Davis, J. 1993*. Finches and sparrows*. C. Helm, A & C, London.

Dunning, J. B. Jr. (ed.) 1993. *CRC Handbook of avian body masses*. CRC Press, Boca Raton, Florida.

Feare, C. and Craig, A. 1998*. Starlings and mynas*. C. Helm, A & C, London

Garamszegi, L. Z., Møller, A. P. and Erritzoe, J. 2002 Coevolving avian eye size and brain size in relation to prey capture and nocturnality. *Proc. R. Soc. London Ser. B* **269**, 961-967.

Harrap, S. and Quinn, D. 1996. *Tits, nutchatches and Treecreepers*. C. Helm, A & C, London.

Pereira, H. M., Daily, G.C. and Roughgarden, J. 2004. A framework for assessing the relative vulnerability of species to land-use change. *Ecol. Appl.* **14**:730–742.

Del Hoyo, J., Elliot, A. and Christie, D.A. (eds.) 2003. *Handbook of the birds of the world. Volume 8: Broadbills to* *Tapaculos.* Barcelona: Lynx Edicions.

Del Hoyo, J., Elliot, A. and Christie, D.A. (eds.) 2004. *Handbook of the birds of the world. Volume 9: Cotingas to Pipits and Wagtails.* Barcelona: Lynx Edicions.

Del Hoyo, J., Elliot, A. and Christie, D.A. (eds.) 2005. *Handbook of the birds of the world. Volume 10: Cuckoo-shrikes to Thrushes.* Barcelona: Lynx Edicions.

Del Hoyo, J., Elliot, A. and Christie, D.A. (eds.) 2006. *Handbook of the birds of the world. Volume 11: Old World Flycatchers to Old World Warblers.* Barcelona: Lynx Edicions.

Del Hoyo, J., Elliot, A. and Christie, D.A. (eds.) 2007. *Handbook of the birds of the world. Volume 11: Picathartes to Tits and Chickadees.* Barcelona: Lynx Edicions.

Del Hoyo, J., Elliot, A. and Christie, D.A. (eds.) 2008. *Handbook of the birds of the world. Volume 12: Penduline-tits to Shrikes.* Barcelona: Lynx Edicions.

Iwaniuk, A. N. 2003 The evolution of brain size and structure in birds. PhD Thesis. Clayton: Monash University.

Iwaniuk, A. N. and Nelson, J. E. 2001 A comparative analysis of relative brain size in waterfowl (Anseriformes). *Brain Behav. Evol.* **57**, 87-97.

Iwaniuk, A. N. and Nelson, J. E. 2002 Can endocranial volume be used as an estimate of brain size in birds? *Can. J. Zool.-Rev. Can. Zool* **80**, 16-23.

Lambert, F. and Woodcock, M. 1996. *Pittas, broadbills and asities*. Pica Press, Sussex.

Madge, S. and Burn, H. 1999. *Crows and jays*. C. Helm, A & C, London

Mlíkovský, J. 1990 Brain size in birds: 4. Passeriformes. *Acta Soc. Zool. Bohemoslov.* **54**, 27-37.

Székely, T., Catchpole, C. K., DeVoogd, A., Marchl, Z. and DeVoogd, T. J. 1996 Evolutionary changes in a song control area of the brain (HVC) are associated with evolutionary changes in song repertoire among European warblers (Sylvidae). *Proc. R. Soc. London Ser. B* **263**, 607-610.
