# Supplementary material for: Evolutionary Divergence in Brain Size between Migratory and Resident Birds
Source: PLoS One. 2010 Mar 10;5(3):e9617. doi: 10.1371/journal.pone.0009617 (PMC2835749; doi:10.1371/journal.pone.0009617)
Supplement: Table S1 — Migratory distance, body mass, brain mass and source of brain data for the species included in this study. Endocranial volumes were converted to mass by multiplying the reported value by the density of fresh brain tissue [1.036 g ml–1, 1]). (0.90 MB DOC) [file pone.0009617.s002.doc]

| **Family** | **Species** | **Migratory distance** | **Body Mass** | **Brain Mass** | **Source of brain data** |
| --- | --- | --- | --- | --- | --- |
| Aegithalidae | *Aegithalos caudatus* | 1 | 8.20 | 0.50 | Published |
| Aegithalidae | *Aegithalos concinnus* | 0 | 6.10 | 0.38 | Published |
| Aegithalidae | *Psaltriparus minimus* | 1 | 5.30 | 0.36 | Endocast |
| Alaudidae | *Eremophila bilopha* | 0 | 23.55 | 0.85 | Endocast |
| Alaudidae | *Galerida cristata* | 0 | 42.13 | 1.08 | Published |
| Alaudidae | *Mirafra javanica* | 1 | 23.00 | 0.76 | Published |
| Bombycillidae | *Dulus dominicus* | 0 | 47.60 | 1.32 | Endocast |
| Certhiidae | *Auriparus flaviceps* | 0 | 6.60 | 0.46 | Endocast |
| Certhiidae | *Campylorhynchus bruneicapillus* | 0 | 38.90 | 1.39 | Endocast |
| Certhiidae | *Certhia familiaris* | 0 | 8.42 | 0.55 | Endocast |
| Certhiidae | *Cyphorhinus aradus* | 0 | 18.70 | 0.94 | Endocast |
| Certhiidae | *Donacobius atricapillus* | 0 | 34.80 | 1.19 | Published |
| Certhiidae | *Henicorhina leucosticta* | 0 | 13.90 | 0.81 | Endocast |
| Certhiidae | *Polioptila dumicola* | 0 | 7.60 | 0.43 | Published |
| Certhiidae | *Ramphocaenus melanurus* | 0 | 9.80 | 0.53 | Published |
| Certhiidae | *Thryomanes bewickii* | 1 | 9.80 | 0.54 | Endocast |
| Certhiidae | *Thryothurus ludovicianus* | 0 | 18.70 | 0.88 | Endocast |
| Cinclidae | *Cinclus mexicanus* | 0 | 57.80 | 1.45 | Published |
| Cisticolidae | *Cisticola cherinus* | 0 | 10.00 | 0.45 | Published |
| Cisticolidae | *Cisticola fulvicapilla* | 0 | 10.30 | 0.53 | Endocast |
| Cisticolidae | *PriniaSchistolais leucopogon* | 0 | 13.80 | 0.61 | Published |
| Climacteridae | *Climacteris picumnus* | 0 | 30.30 | 1.04 | Endocast |
| Climacteridae | *Climacteris rufa* | 0 | 33.30 | 1.07 | Endocast |
| Climacteridae | *Cormobates leucophaeus* | 0 | 22.40 | 0.84 | Endocast |
| Conopophagidae | *Conopophaga lineata* | 0 | 21.80 | 0.73 | Endocast |
| Corvidae | *Aegithina tiphia* | 0 | 12.30 | 0.69 | Endocast |
| Corvidae | *Aphelocoma caerulescens* | 0 | 76.00 | 2.95 | Endocast |
| Corvidae | *Aphelocoma ultramarina* | 0 | 128.40 | 3.74 | Published |
| Corvidae | *Artamus cinereus* | 0 | 38.00 | 0.95 | Endocast |
| Corvidae | *Artamus cyanopterus* | 0 | 35.50 | 1.04 | Endocast |
| Corvidae | *Artamus leucorhynchus* | 1 | 45.60 | 1.12 | Endocast |
| Corvidae | *Artamus superciliosus* | 1 | 39.20 | 1.08 | Endocast |
| Corvidae | *Batis capensis* | 1 | 12.80 | 0.70 | Endocast |
| Corvidae | *Bias musicus* | 1 | 21.70 | 0.87 | Published |
| Corvidae | *Campephaga phoenicea* | 0 | 28.50 | 1.09 | Endocast |
| Corvidae | *Cicinnurus regius* | 0 | 52.00 | 1.80 | Endocast |
| Corvidae | *Cinclosoma cinnamomeum* | 0 | 58.60 | 1.52 | Published |
| Corvidae | *Cissa chinensis* | 0 | 106.50 | 4.33 | Published |
| Corvidae | *Colluricincla harmonica* | 0 | 63.30 | 2.07 | Endocast |
| Corvidae | *Coracina caledonica* | 0 | 145.30 | 3.10 | Published |
| Corvidae | *Coracina novaehollandiae* | 1 | 127.70 | 2.49 | Endocast |
| Corvidae | *Corcorax melanorhamphus* | 1 | 349.10 | 5.39 | Endocast |
| Corvidae | *Corvus albicollis* | 0 | 900.00 | 12.00 | Published |
| Corvidae | *Corvus albus* | 0 | 584.10 | 9.07 | Endocast |
| Corvidae | *Corvus brachyrhynchos* | 4 | 438.50 | 7.43 | Endocast |
| Corvidae | *Corvus corax* | 0 | 1405.00 | 15.31 | Endocast |
| Corvidae | *Corvus corone* | 4 | 523.95 | 7.36 | Endocast |
| Corvidae | *Corvus coronoides* | 0 | 675.00 | 10.18 | Endocast |
| Corvidae | *Corvus cryptoleucos* | 2 | 534.00 | 9.27 | Endocast |
| Corvidae | *Corvus frugilegus* | 3 | 509.30 | 8.13 | Endocast |
| Corvidae | *Corvus mellori* | 0 | 300.00 | 8.81 | Endocast |
| Corvidae | *Corvus monedula* | 2 | 211.29 | 4.69 | Endocast |
| Corvidae | *Corvus orru* | 0 | 522.20 | 9.23 | Endocast |
| Corvidae | *Corvus ossifragus* | 0 | 285.00 | 6.21 | Endocast |
| Corvidae | *Cracticus torquatus* | 0 | 104.10 | 2.88 | Published |
| Corvidae | *Cyanocitta cristata* | 3 | 89.10 | 3.03 | Endocast |
| Corvidae | *Cyanocitta stelleri* | 0 | 128.00 | 3.67 | Endocast |
| Corvidae | *Cyanocorax chrysops* | 0 | 157.00 | 4.30 | Published |
| Corvidae | *Cyanocorax yncas* | 0 | 78.50 | 2.40 | Published |
| Corvidae | *Cyanopica cyana* | 0 | 72.00 | 3.00 | Published |
| Corvidae | *Dendrocitta vagabunda* | 0 | 100.00 | 2.80 | Published |
| Corvidae | *Dryoscopus cubla* | 0 | 26.40 | 1.17 | Endocast |
| Corvidae | *Falculuncus frontatus* | 1 | 26.10 | 1.28 | Endocast |
| Corvidae | *Garrulus glandarius* | 0 | 168.89 | 4.15 | Published |
| Corvidae | *Grallina cyanoleuca* | 0 | 89.00 | 1.74 | Endocast |
| Corvidae | *Gymnorhina tibicen* | 0 | 314.00 | 4.82 | Endocast |
| Corvidae | *Gymnorhinus cyanocephalus* | 1 | 103.00 | 3.64 | Endocast |
| Corvidae | *Hypothymis azurea* | 0 | 10.20 | 0.54 | Endocast |
| Corvidae | *Lalage sueurii* | 0 | 18.90 | 0.90 | Published |
| Corvidae | *Laniarius erythrogaster* | 0 | 45.80 | 1.62 | Published |
| Corvidae | *Lophorina superba* | 0 | 78.00 | 2.69 | Published |
| Corvidae | *Monarcha guttula* | 0 | 15.50 | 0.70 | Endocast |
| Corvidae | *Monarcha melanopsis* | 1 | 20.20 | 0.95 | Endocast |
| Corvidae | *Monarcha trivirgatus* | 1 | 13.50 | 0.65 | Endocast |
| Corvidae | *Myiagra alecto* | 0 | 24.00 | 0.76 | Endocast |
| Corvidae | *Myiagra caledonica* | 0 | 11.00 | 0.61 | Endocast |
| Corvidae | *Myiagra inquieta* | 1 | 14.30 | 0.79 | Endocast |
| Corvidae | *Nucifraga caryocatactes* | 0 | 189.47 | 5.71 | Published |
| Corvidae | *Oreoica gutturalis* | 0 | 62.00 | 1.73 | Endocast |
| Corvidae | *Oriolus oriolus* | 4 | 71.90 | 1.50 | Published |
| Corvidae | *Parotia lawesii* | 0 | 156.50 | 4.15 | Endocast |
| Corvidae | *Pericrocotus ethologus* | 1 | 18.00 | 0.91 | Endocast |
| Corvidae | *Perisoreus canadensis* | 0 | 69.00 | 2.62 | Endocast |
| Corvidae | *Pica pica* | 0 | 206.13 | 5.34 | Endocast |
| Corvidae | *Pitohui ferrugineus* | 0 | 94.00 | 2.57 | Published |
| Corvidae | *Platysteira cyanea* | 0 | 11.84 | 0.60 | Published |
| Corvidae | *Podoce hendersoni* | 0 | 149.00 | 3.40 | Published |
| Corvidae | *Prionops plumatus* | 1 | 35.50 | 1.20 | Endocast |
| Corvidae | *Psilorhinus morio* | 0 | 204.00 | 4.97 | Published |
| Corvidae | *Psophodes occidentalis* | 0 | 42.00 | 1.38 | Published |
| Corvidae | *Ptiloris paradiseus* | 0 | 125.50 | 2.88 | Endocast |
| Corvidae | *Ptiloris victoriae* | 0 | 92.00 | 2.63 | Endocast |
| Corvidae | *Pyrrhocorax graculus* | 1 | 223.50 | 3.20 | Published |
| Corvidae | *Pyrrhocorax pyrrhocorax* | 1 | 324.00 | 6.61 | Published |
| Corvidae | *Rhipidura leucophrys* | 1 | 27.70 | 0.60 | Published |
| Corvidae | *Rhipidura rufifrons* | 1 | 10.20 | 0.37 | Endocast |
| Corvidae | *Sphecotheres viridis* | 1 | 132.40 | 2.32 | Endocast |
| Corvidae | *Strepera fuliginosa* | 0 | 300.00 | 5.91 | Endocast |
| Corvidae | *Struthidea cinerea* | 1 | 134.30 | 3.09 | Endocast |
| Corvidae | *Tchagra australis* | 0 | 36.30 | 1.27 | Published |
| Corvidae | *Telophorus zeylonus* | 0 | 62.70 | 1.98 | Endocast |
| Corvidae | *Tephrodornis pondicerianus* | 0 | 19.50 | 0.92 | Published |
| Corvidae | *Urocissa erythrorhyncha* | 1 | 214.00 | 3.80 | Published |
| Eurylaimidae | *Smithornis capensis* | 0 | 25.50 | 0.81 | Published |
| Formicariidae | *Chamaeza campanisona* | 0 | 97.20 | 1.90 | Published |
| Formicariidae | *Formicarius analis* | 0 | 54.20 | 1.35 | Endocast |
| Formicariidae | *Formicarius colma* | 0 | 45.30 | 1.12 | Endocast |
| Formicariidae | *Grallaria quitensis* | 0 | 79.50 | 2.12 | Endocast |
| Fringillidae | *Agelaius phoeniceus* | 3 | 65.50 | 1.75 | Endocast |
| Fringillidae | *Agelaius ruficapillus* | 1 | 36.75 | 0.81 | Published |
| Fringillidae | *Aimophila cassinii* | 2 | 18.10 | 0.81 | Endocast |
| Fringillidae | *Amblycercus holosericeus* | 0 | 66.90 | 2.29 | Endocast |
| Fringillidae | *Amblyramphus holosericeus* | 0 | 69.00 | 2.41 | Endocast |
| Fringillidae | *Ammodramus savannarum* | 4 | 17.00 | 0.67 | Published |
| Fringillidae | *Amphispiza bilineata* | 3 | 13.80 | 0.70 | Endocast |
| Fringillidae | *Anisognathus somptuosus* | 0 | 42.00 | 1.54 | Published |
| Fringillidae | *Arremonops conirostris* | 0 | 37.30 | 1.34 | Published |
| Fringillidae | *Basileuterus culicivorus* | 0 | 8.90 | 0.60 | Endocast |
| Fringillidae | *Basileuterus fulvicauda* | 0 | 14.90 | 0.58 | Published |
| Fringillidae | *BuarremonAtlapetes brunneinucha* | 0 | 46.60 | 1.60 | Endocast |
| Fringillidae | *Cacicus cela* | 0 | 91.50 | 2.52 | Published |
| Fringillidae | *Calamospiza melanocorys* | 4 | 37.80 | 1.22 | Endocast |
| Fringillidae | *Calcarius lapponicus* | 4 | 27.50 | 0.90 | Endocast |
| Fringillidae | *Cardellina rubrifrons* | 0 | 9.80 | 0.45 | Endocast |
| Fringillidae | *Cardinalis cardinalis* | 0 | 44.10 | 1.57 | Published |
| Fringillidae | *Carduelis ambigua* | 1 | 14.10 | 0.66 | Published |
| Fringillidae | *Carduelis cannabina* | 3 | 18.78 | 0.67 | Published |
| Fringillidae | *Carduelis carduelis* | 3 | 15.00 | 0.59 | Endocast |
| Fringillidae | *Carduelis chloris* | 2 | 26.50 | 0.89 | Published |
| Fringillidae | *Carduelis flammea* | 3 | 12.13 | 0.60 | Endocast |
| Fringillidae | *Carduelis pinus* | 2 | 14.60 | 0.59 | Endocast |
| Fringillidae | *Carduelis spinus* | 2 | 12.08 | 0.56 | Published |
| Fringillidae | *Carduelis tristis* | 3 | 12.90 | 0.56 | Published |
| Fringillidae | *Carpodacus mexicanus* | 0 | 20.40 | 0.81 | Endocast |
| Fringillidae | *Carpodacus purpureus* | 2 | 25.60 | 0.93 | Endocast |
| Fringillidae | *Carpodacus roseus* | 4 | 21.15 | 1.01 | Published |
| Fringillidae | *Caryothraustes poliogaster* | 0 | 37.50 | 1.48 | Published |
| Fringillidae | *Chlorophonia cyanea* | 0 | 14.00 | 0.63 | Published |
| Fringillidae | *Chlorospingus ophthalmicus* | 0 | 19.00 | 1.02 | Endocast |
| Fringillidae | *Chlorothraupis carmioli* | 0 | 39.00 | 1.35 | Published |
| Fringillidae | *Cissopis leveriana* | 0 | 76.00 | 1.77 | Published |
| Fringillidae | *Coccothraustes coccothraustes* | 0 | 54.00 | 1.63 | Published |
| Fringillidae | *Coereba flaveola* | 0 | 8.50 | 0.46 | Endocast |
| Fringillidae | *Coryphospingus cucullatus* | 0 | 14.60 | 0.69 | Endocast |
| Fringillidae | *Curaeus curaeus* | 0 | 90.00 | 2.56 | Published |
| Fringillidae | *Cyanerpes cyaneus* | 0 | 14.00 | 0.57 | Endocast |
| Fringillidae | *Dacnis cayana* | 0 | 13.00 | 0.57 | Published |
| Fringillidae | *Dendroica caerulescens* | 4 | 9.40 | 0.45 | Endocast |
| Fringillidae | *Dendroica castanea* | 4 | 12.00 | 0.41 | Published |
| Fringillidae | *Dendroica magnolia* | 4 | 8.70 | 0.38 | Published |
| Fringillidae | *Dendroica pensylvanica* | 4 | 9.40 | 0.39 | Published |
| Fringillidae | *Dendroica petechia* | 4 | 9.80 | 0.54 | Endocast |
| Fringillidae | *Dives dives* | 0 | 96.20 | 2.28 | Published |
| Fringillidae | *Dolichonyx oryzivorus* | 4 | 38.70 | 1.11 | Published |
| Fringillidae | *Emberiza cirlus* | 1 | 23.10 | 0.79 | Published |
| Fringillidae | *Emberiza citrinella* | 3 | 28.58 | 0.82 | Published |
| Fringillidae | *Emberiza elegans* | 3 | 20.50 | 0.64 | Published |
| Fringillidae | *Emberiza rutilla* | 4 | 17.18 | 0.59 | Published |
| Fringillidae | *Emberiza schoeniclus* | 4 | 20.57 | 0.68 | Published |
| Fringillidae | *Emberiza spodocephala* | 4 | 18.00 | 0.96 | Published |
| Fringillidae | *Euneornis campestris* | 0 | 16.20 | 0.73 | Endocast |
| Fringillidae | *Euphagus carolinus* | 4 | 55.70 | 1.56 | Endocast |
| Fringillidae | *Euphagus cyanocephalus* | 3 | 68.50 | 1.49 | Endocast |
| Fringillidae | *Euphonia jamaica* | 0 | 16.10 | 0.67 | Published |
| Fringillidae | *Fringilla coelebs* | 4 | 24.26 | 0.77 | Endocast |
| Fringillidae | *Fringilla montifringilla* | 3 | 24.94 | 0.78 | Published |
| Fringillidae | *Gnorimopsar chopi* | 1 | 79.50 | 1.86 | Published |
| Fringillidae | *Gymnostinops montezuma* | 0 | 376.50 | 6.10 | Endocast |
| Fringillidae | *Habia fuscicauda* | 1 | 39.70 | 1.39 | Published |
| Fringillidae | *Helmitheros vermivorus* | 4 | 14.20 | 0.61 | Endocast |
| Fringillidae | *Hemithraupis guira* | 0 | 12.00 | 0.59 | Published |
| Fringillidae | *Himatione sanguinea* | 0 | 14.80 | 0.69 | Published |
| Fringillidae | *Icteria virens* | 4 | 24.90 | 0.94 | Published |
| Fringillidae | *Icterus galbula* | 4 | 33.30 | 1.17 | Endocast |
| Fringillidae | *Icterus spurius* | 4 | 20.40 | 0.89 | Endocast |
| Fringillidae | *Junco hyemalis* | 4 | 19.00 | 0.86 | Endocast |
| Fringillidae | *Leucosticte arctoa* | 0 | 25.60 | 1.36 | Published |
| Fringillidae | *Limnothlypis swainsonii* | 3 | 14.00 | 0.69 | Endocast |
| Fringillidae | *Loxia curvirostra* | 0 | 34.30 | 1.47 | Endocast |
| Fringillidae | *Loxia leucoptera* | 0 | 31.80 | 1.43 | Endocast |
| Fringillidae | *Loxigilla violacea* | 0 | 28.00 | 1.20 | Endocast |
| Fringillidae | *Loxipasser anoxanthus* | 0 | 11.30 | 0.59 | Endocast |
| Fringillidae | *Melopyrrha nigra* | 0 | 10.90 | 0.87 | Endocast |
| Fringillidae | *Melospiza georgiana* | 4 | 17.60 | 0.84 | Published |
| Fringillidae | *Melospiza melodia* | 4 | 20.50 | 0.96 | Published |
| Fringillidae | *Mitrospingus cassinii* | 0 | 40.40 | 1.36 | Published |
| Fringillidae | *Mniotilta varia* | 4 | 12.00 | 0.46 | Endocast |
| Fringillidae | *Molothrus ater* | 3 | 41.70 | 1.19 | Endocast |
| Fringillidae | *Molothrus badius* | 0 | 44.50 | 1.53 | Endocast |
| Fringillidae | *Myioborus pictus* | 0 | 9.80 | 0.40 | Endocast |
| Fringillidae | *Nesospingus speculiferus* | 0 | 36.20 | 1.48 | Endocast |
| Fringillidae | *Paroaria coronata* | 0 | 43.00 | 1.27 | Published |
| Fringillidae | *Passerculus sandwichensis* | 4 | 20.10 | 0.70 | Published |
| Fringillidae | *Passerina cyanea* | 4 | 12.20 | 0.67 | Endocast |
| Fringillidae | *Phaenocophilus palmarum* | 0 | 29.20 | 1.17 | Endocast |
| Fringillidae | *Pheucticus ludovicianus* | 4 | 45.60 | 1.37 | Endocast |
| Fringillidae | *Pinicola enucleator* | 0 | 65.70 | 1.64 | Endocast |
| Fringillidae | *Pipilo erythrophthalmus* | 2 | 39.20 | 1.41 | Endocast |
| Fringillidae | *Protonotaria citrea* | 4 | 15.50 | 0.61 | Published |
| Fringillidae | *Pseudoleistes virescens* | 0 | 70.40 | 2.08 | Endocast |
| Fringillidae | *Pyrrhula Pyrrhula* | 2 | 21.80 | 0.97 | Published |
| Fringillidae | *Quiscalus major* | 0 | 158.80 | 2.88 | Endocast |
| Fringillidae | *Quiscalus mexicanus* | 0 | 168.70 | 3.07 | Endocast |
| Fringillidae | *Quiscalus quiscula* | 4 | 110.20 | 2.68 | Endocast |
| Fringillidae | *Rhodinocichla rosea* | 0 | 46.70 | 1.40 | Published |
| Fringillidae | *Rhodospingus cruentus* | 0 | 10.80 | 0.54 | Published |
| Fringillidae | *Saltator maximus* | 0 | 46.60 | 1.44 | Endocast |
| Fringillidae | *Scaphidura oryzivora* | 0 | 190.50 | 3.25 | Endocast |
| Fringillidae | *Schistochlamys melanopis* | 0 | 33.00 | 1.26 | Published |
| Fringillidae | *Seiurus aurocapilla* | 4 | 22.10 | 0.73 | Endocast |
| Fringillidae | *Serinus burtoni* | 0 | 29.60 | 1.21 | Published |
| Fringillidae | *Serinus canaria* | 0 | 8.40 | 0.45 | Published |
| Fringillidae | *Serinus flaviventris* | 0 | 16.30 | 0.75 | Endocast |
| Fringillidae | *Serinus serinus* | 3 | 11.20 | 0.65 | Published |
| Fringillidae | *Setophaga ruticilla* | 4 | 7.80 | 0.37 | Endocast |
| Fringillidae | *Sicalis flaveola* | 0 | 19.70 | 0.73 | Published |
| Fringillidae | *Spindalis zena* | 0 | 42.50 | 1.36 | Endocast |
| Fringillidae | *Spiza americana* | 4 | 27.00 | 0.94 | Endocast |
| Fringillidae | *Spizella arborea* | 4 | 12.40 | 0.77 | Published |
| Fringillidae | *Sporophila americana* | 0 | 10.70 | 0.62 | Published |
| Fringillidae | *Sturnella magna* | 3 | 89.00 | 2.30 | Published |
| Fringillidae | *Sturnella neglecta* | 3 | 103.50 | 2.01 | Endocast |
| Fringillidae | *Tachyphonus delattrii* | 0 | 16.80 | 0.77 | Published |
| Fringillidae | *Tangara cyanicollis* | 0 | 17.00 | 0.66 | Published |
| Fringillidae | *Tangara icterocephala* | 0 | 15.00 | 0.59 | Published |
| Fringillidae | *Thraupis episcopus* | 0 | 31.10 | 1.15 | Endocast |
| Fringillidae | *Tiaris olivacea* | 0 | 8.50 | 0.50 | Endocast |
| Fringillidae | *Vermivora peregrina* | 4 | 9.50 | 0.45 | Endocast |
| Fringillidae | *Vermivora pinus* | 4 | 8.40 | 0.42 | Published |
| Fringillidae | *Volatinia jacarina* | 1 | 12.50 | 0.51 | Endocast |
| Fringillidae | *Xanthocephalus xanthocephalus* | 4 | 76.60 | 1.71 | Endocast |
| Fringillidae | *Zonotrichia albicollis* | 4 | 27.40 | 1.09 | Endocast |
| Fringillidae | *Zonotrichia capensis* | 1 | 20.30 | 0.80 | Published |
| Fringillidae | *Zonotrichia leucophrys* | 2 | 26.40 | 1.00 | Endocast |
| Furnariidae | *Anumbius annumbi* | 0 | 41.50 | 1.22 | Endocast |
| Furnariidae | *Aphrastura spinicauda* | 0 | 11.50 | 0.84 | Endocast |
| Furnariidae | *Automolus infuscatus* | 0 | 33.10 | 1.17 | Endocast |
| Furnariidae | *Automolus ochrolaemus* | 0 | 40.20 | 1.18 | Endocast |
| Furnariidae | *Campylorhamphus pusillus* | 1 | 43.30 | 1.39 | Endocast |
| Furnariidae | *Certhiaxis cinnamomea* | 0 | 14.80 | 0.71 | Endocast |
| Furnariidae | *Cinclodes fuscus* | 1 | 30.45 | 0.93 | Endocast |
| Furnariidae | *Cinclodes patagonicus* | 1 | 53.20 | 1.28 | Endocast |
| Furnariidae | *Coryphistera alaudina* | 0 | 34.30 | 0.97 | Endocast |
| Furnariidae | *Dendrocincla fuliginosa* | 1 | 39.40 | 1.10 | Endocast |
| Furnariidae | *Dendrocolaptes certhia* | 0 | 66.70 | 1.80 | Endocast |
| Furnariidae | *Furnarius rufus* | 0 | 63.70 | 1.35 | Endocast |
| Furnariidae | *Glyphorhynchus spirurus* | 1 | 14.80 | 0.64 | Endocast |
| Furnariidae | *Lepidocolaptes affinis* | 1 | 29.00 | 1.17 | Published |
| Furnariidae | *Lepidocolaptes souleyetii* | 0 | 25.70 | 1.08 | Published |
| Furnariidae | *Phacellodomus ruber* | 0 | 39.30 | 1.15 | Endocast |
| Furnariidae | *Phleocryptes melanops* | 1 | 14.20 | 0.71 | Endocast |
| Furnariidae | *Sclerurus mexicanus* | 0 | 21.10 | 0.82 | Endocast |
| Furnariidae | *Sittasomus griseicapillus* | 0 | 14.00 | 0.57 | Endocast |
| Furnariidae | *Xenops minutus* | 0 | 11.70 | 0.51 | Endocast |
| Furnariidae | *Xiphorhynchus guttatus* | 0 | 46.40 | 1.55 | Endocast |
| Furnariidae | *Xiphorhynchus picus* | 0 | 34.60 | 1.35 | Endocast |
| Hirundinidae | *Delichon urbica* | 4 | 14.50 | 0.50 | Published |
| Hirundinidae | *Hirundo rustica* | 4 | 17.54 | 0.58 | Endocast |
| Hirundinidae | *Notiochelidon cyanoleuca* | 4 | 9.70 | 0.45 | Published |
| Hirundinidae | *Progne subis* | 4 | 50.70 | 1.07 | Endocast |
| Hirundinidae | *Riparia riparia* | 4 | 13.80 | 0.42 | Endocast |
| Hirundinidae | *Stelgidopteryx ruficollis* | 3 | 15.20 | 0.55 | Published |
| Hirundinidae | *Tachycineta bicolor* | 4 | 20.10 | 0.57 | Endocast |
| Hirundinidae | *Tachycineta thalassina* | 4 | 15.10 | 0.48 | Endocast |
| Irenidae | *Chloropsis hardwickii* | 1 | 23.75 | 0.76 | Published |
| Irenidae | *Chloropsis palawanensis* | 0 | 30.50 | 1.09 | Published |
| Irenidae | *Irena puella* | 0 | 58.37 | 1.12 | Endocast |
| Laniidae | *Lanius collaris* | 0 | 35.85 | 1.11 | Published |
| Laniidae | *Lanius collurio* | 4 | 29.90 | 0.99 | Endocast |
| Laniidae | *Lanius excubitor* | 3 | 59.63 | 1.48 | Published |
| Laniidae | *Lanius ludovicianus* | 4 | 47.40 | 1.59 | Endocast |
| Laniidae | *Lanius senator* | 4 | 27.80 | 1.11 | Endocast |
| Maluridae | *Amytornis goyderi* | 0 | 16.70 | 0.81 | Published |
| Maluridae | *Malurus cyaneus* | 0 | 8.30 | 0.49 | Endocast |
| Maluridae | *Malurus elegans* | 0 | 10.10 | 0.59 | Endocast |
| Maluridae | *Malurus lamberti* | 0 | 9.20 | 0.48 | Endocast |
| Maluridae | *Malurus leucopterus* | 0 | 8.00 | 0.40 | Endocast |
| Maluridae | *Malurus melanocephalus* | 0 | 7.00 | 0.47 | Endocast |
| Maluridae | *Malurus pulcherrimus* | 0 | 9.80 | 0.44 | Endocast |
| Maluridae | *Malurus splendens* | 0 | 11.40 | 0.47 | Endocast |
| Melanocharitidae | *Toxorhamphus iliolophum* | 0 | 12.90 | 0.50 | Published |
| Melanocharitidae | *Toxorhamphus poliopterus* | 0 | 11.40 | 0.45 | Endocast |
| Meliphagidae | *Acanthagenys rufogularis* | 0 | 50.20 | 1.20 | Published |
| Meliphagidae | *Acanthorynchus tenuirostris* | 1 | 11.20 | 0.50 | Endocast |
| Meliphagidae | *Anthochaera carunculata* | 1 | 108.50 | 2.28 | Endocast |
| Meliphagidae | *Anthochaera chrysoptera* | 1 | 66.30 | 1.76 | Endocast |
| Meliphagidae | *Ashbyia lovensis* | 0 | 17.50 | 0.69 | Published |
| Meliphagidae | *Conopophila rufogularis* | 1 | 10.80 | 0.46 | Published |
| Meliphagidae | *Entomyzon cyanotis* | 1 | 106.50 | 2.31 | Endocast |
| Meliphagidae | *Epthianura aurifrons* | 1 | 10.30 | 0.45 | Endocast |
| Meliphagidae | *Epthianura tricolor* | 1 | 10.70 | 0.46 | Endocast |
| Meliphagidae | *Lichenostomus chrysops* | 1 | 17.50 | 0.75 | Endocast |
| Meliphagidae | *Lichenostomus flavescens* | 1 | 12.60 | 0.54 | Endocast |
| Meliphagidae | *Lichenostomus flavus* | 0 | 21.10 | 0.74 | Endocast |
| Meliphagidae | *Lichenostomus keartlandi* | 1 | 15.30 | 0.71 | Endocast |
| Meliphagidae | *Lichenostomus leucotis* | 1 | 22.10 | 0.92 | Endocast |
| Meliphagidae | *Lichenostomus melanops* | 1 | 19.80 | 0.95 | Endocast |
| Meliphagidae | *Lichenostomus ornatus* | 1 | 19.80 | 0.78 | Endocast |
| Meliphagidae | *Lichenostomus penicillatus* | 1 | 19.20 | 0.86 | Endocast |
| Meliphagidae | *Lichenostomus virescens* | 1 | 33.30 | 0.95 | Endocast |
| Meliphagidae | *Lichmera indistincta* | 1 | 11.40 | 0.53 | Endocast |
| Meliphagidae | *Manorina flavigula* | 0 | 59.80 | 1.42 | Endocast |
| Meliphagidae | *Manorina melanocephala* | 0 | 60.90 | 1.93 | Endocast |
| Meliphagidae | *Manorina melanophrys* | 0 | 25.50 | 1.18 | Endocast |
| Meliphagidae | *Meliphaga lewinii* | 1 | 36.20 | 1.21 | Endocast |
| Meliphagidae | *Meliphaga notata* | 1 | 26.40 | 0.90 | Endocast |
| Meliphagidae | *Melithreptus albogularis* | 1 | 11.10 | 0.65 | Endocast |
| Meliphagidae | *Melithreptus brevirostris* | 1 | 14.30 | 0.70 | Endocast |
| Meliphagidae | *Melithreptus lunatus* | 1 | 14.10 | 0.62 | Endocast |
| Meliphagidae | *Myzomela obscura* | 1 | 11.90 | 0.53 | Endocast |
| Meliphagidae | *Myzomela sanguinolenta* | 1 | 7.80 | 0.35 | Endocast |
| Meliphagidae | *Philemon buceroides* | 1 | 121.00 | 2.23 | Endocast |
| Meliphagidae | *Philemon corniculatus* | 1 | 105.80 | 2.05 | Endocast |
| Meliphagidae | *Phylidonyris albifrons* | 1 | 17.10 | 0.75 | Published |
| Meliphagidae | *Phylidonyris melanops* | 1 | 18.30 | 0.64 | Published |
| Meliphagidae | *Phylidonyris novaehollandiae* | 1 | 19.40 | 0.93 | Published |
| Meliphagidae | *Ramsayornis modestus* | 0 | 10.60 | 0.53 | Endocast |
| Meliphagidae | *Xanthotis flaviventer* | 1 | 33.60 | 1.09 | Endocast |
| Menuridae | *Menura novaehollandiae* | 0 | 644.40 | 11.11 | Endocast |
| Muscicapidae | *Alethe diademata* | 0 | 33.60 | 1.08 | Published |
| Muscicapidae | *Brachypteryx montana* | 1 | 18.00 | 0.86 | Endocast |
| Muscicapidae | *Catharus fuscescens* | 4 | 41.50 | 0.95 | Endocast |
| Muscicapidae | *Catharus minimus* | 4 | 32.80 | 0.80 | Published |
| Muscicapidae | *Catharus ustulatus* | 4 | 30.80 | 0.82 | Published |
| Muscicapidae | *Cercotrichas coryphaeus* | 0 | 23.10 | 0.75 | Endocast |
| Muscicapidae | *Cichlerminia lherminieri* | 0 | 100.00 | 2.28 | Endocast |
| Muscicapidae | *Copsychus malabaricus* | 0 | 30.00 | 0.90 | Published |
| Muscicapidae | *Copsychus sauIaris* | 0 | 33.20 | 1.14 | Published |
| Muscicapidae | *Cossypha caffra* | 1 | 28.50 | 1.06 | Endocast |
| Muscicapidae | *Culicicapa helianthea* | 0 | 7.50 | 0.32 | Endocast |
| Muscicapidae | *Enicurus scouleri* | 1 | 14.00 | 0.79 | Endocast |
| Muscicapidae | *Erithacus rubecula* | 1 | 16.75 | 0.66 | Published |
| Muscicapidae | *Hylocichla mustelinus* | 4 | 47.40 | 1.30 | Endocast |
| Muscicapidae | *Monticola saxatilis* | 4 | 48.50 | 1.34 | Published |
| Muscicapidae | *Myadestes genibarbis* | 1 | 27.10 | 0.95 | Endocast |
| Muscicapidae | *Myadestes townsendi* | 4 | 32.50 | 1.05 | Endocast |
| Muscicapidae | *Phoenicurus ochruros* | 1 | 16.50 | 0.70 | Endocast |
| Muscicapidae | *Rhinomyias gularis* | 0 | 18.00 | 1.09 | Published |
| Muscicapidae | *Saxicola torquata* | 1 | 15.30 | 0.63 | Endocast |
| Muscicapidae | *Sialia mexicana* | 1 | 26.40 | 0.87 | Endocast |
| Muscicapidae | *Sigelus silens* | 0 | 28.30 | 1.04 | Published |
| Muscicapidae | *Turdus merula* | 3 | 98.17 | 1.92 | Endocast |
| Muscicapidae | *Turdus migratorius* | 4 | 80.20 | 1.70 | Endocast |
| Muscicapidae | *Turdus philomelos* | 4 | 69.64 | 1.59 | Endocast |
| Muscicapidae | *Zoothera lunulata* | 0 | 104.00 | 2.24 | Endocast |
| Nectariniidae | *Aethopyga nipalensis* | 1 | 6.00 | 0.34 | Published |
| Nectariniidae | *Anthreptes malacensis* | 0 | 11.90 | 0.60 | Endocast |
| Nectariniidae | *Arachnothera longirostra* | 1 | 11.70 | 0.54 | Endocast |
| Nectariniidae | *Dicaeum aeruginosum* | 1 | 11.80 | 0.52 | Published |
| Nectariniidae | *Dicaeum hirundinaceum* | 1 | 8.00 | 0.38 | Endocast |
| Nectariniidae | *Nectarinia jugularis* | 1 | 8.70 | 0.41 | Endocast |
| Nectariniidae | *Nectarinia verticalis* | 0 | 14.00 | 0.52 | Published |
| Nectariniidae | *Prionochilus plateri* | 0 | 7.90 | 0.41 | Endocast |
| Orthonychidae | *Orthonyx temminckii* | 0 | 62.30 | 1.82 | Endocast |
| Pardalotidae | *Acanthiza chrysorrhoa* | 1 | 10.00 | 0.45 | Endocast |
| Pardalotidae | *Acanthiza lineata* | 0 | 6.40 | 0.53 | Endocast |
| Pardalotidae | *Acanthiza pusilla* | 1 | 6.00 | 0.47 | Endocast |
| Pardalotidae | *Acanthiza reguloides* | 0 | 7.50 | 0.40 | Endocast |
| Pardalotidae | *Acanthiza uropygialis* | 0 | 6.50 | 0.37 | Endocast |
| Pardalotidae | *Aphelocephala leucopsis* | 0 | 12.90 | 0.56 | Endocast |
| Pardalotidae | *Dasyornis broadbenti* | 0 | 83.30 | 1.95 | Endocast |
| Pardalotidae | *Pardalotus punctatus* | 1 | 9.20 | 0.41 | Endocast |
| Pardalotidae | *Pardalotus striatus* | 1 | 11.60 | 0.54 | Endocast |
| Pardalotidae | *Sericornis citreogularis* | 0 | 16.60 | 0.82 | Endocast |
| Pardalotidae | *Sericornis magnirostris* | 0 | 8.50 | 0.62 | Endocast |
| Paridae | *Baeolophus bicolor* | 0 | 21.60 | 1.05 | Endocast |
| Paridae | *Parus ater* | 1 | 8.00 | 0.51 | Published |
| Paridae | *Parus caeruleus* | 1 | 10.79 | 0.65 | Published |
| Paridae | *Parus cristatus* | 0 | 10.20 | 0.70 | Published |
| Paridae | *Parus cyanus* | 0 | 9.95 | 0.66 | Published |
| Paridae | *Parus major* | 0 | 17.02 | 0.85 | Published |
| Paridae | *Parus montanus* | 1 | 10.20 | 0.79 | Published |
| Paridae | *Parus palustris* | 1 | 10.60 | 0.58 | Published |
| Paridae | *Parus venustulus* | 0 | 10.54 | 0.53 | Published |
| Paridae | *Poecile atricapillus* | 0 | 12.00 | 0.79 | Endocast |
| Paridae | *Poecile carolinensis* | 0 | 10.20 | 0.62 | Published |
| Paridae | *Poecile gambeli* | 0 | 11.30 | 0.78 | Endocast |
| Paridae | *Poecile hudsonicus* | 0 | 11.00 | 0.74 | Endocast |
| Passeridae | *Amadina fasciata* | 0 | 15.40 | 0.61 | Published |
| Passeridae | *Amandava amandava* | 4 | 9.60 | 0.39 | Endocast |
| Passeridae | *Anthus berthelotii* | 0 | 15.99 | 0.52 | Published |
| Passeridae | *Anthus campestris* | 4 | 23.00 | 0.48 | Published |
| Passeridae | *Anthus novaeseelandiae* | 1 | 27.90 | 0.87 | Published |
| Passeridae | *Anthus pratensis* | 4 | 18.40 | 0.53 | Published |
| Passeridae | *Anthus trivialis* | 4 | 18.40 | 0.62 | Endocast |
| Passeridae | *Bubalornis albirostris* | 0 | 64.50 | 2.21 | Published |
| Passeridae | *Chloebia gouldiae* | 2 | 10.00 | 0.55 | Published |
| Passeridae | *Erythrura trichroa* | 1 | 14.40 | 0.60 | Endocast |
| Passeridae | *Estrilda astrild* | 1 | 7.50 | 0.35 | Published |
| Passeridae | *Estrilda troglodytes* | 0 | 6.10 | 0.36 | Published |
| Passeridae | *Euplectes orix* | 1 | 16.30 | 0.79 | Published |
| Passeridae | *Foudia madagascariensis* | 0 | 16.00 | 0.78 | Endocast |
| Passeridae | *Lagonosticta senegala* | 1 | 8.30 | 0.40 | Published |
| Passeridae | *Lonchura bicolor* | 0 | 9.69 | 0.44 | Published |
| Passeridae | *Lonchura cucullata* | 1 | 9.20 | 0.39 | Endocast |
| Passeridae | *Lonchura flaviprymna* | 1 | 11.68 | 0.43 | Published |
| Passeridae | *Lonchura malacca* | 0 | 12.60 | 0.62 | Endocast |
| Passeridae | *Lonchura pallida* | 0 | 10.65 | 0.52 | Published |
| Passeridae | *Lonchura spectabilis* | 0 | 7.90 | 0.49 | Published |
| Passeridae | *Lonchura striata* | 1 | 12.30 | 0.48 | Published |
| Passeridae | *Montifringilla nivalis* | 1 | 36.90 | 1.09 | Published |
| Passeridae | *Motacilla alba* | 4 | 18.96 | 0.58 | Published |
| Passeridae | *Motacilla flava* | 4 | 14.40 | 0.57 | Published |
| Passeridae | *Neochmia phaethon* | 0 | 10.00 | 0.52 | Endocast |
| Passeridae | *Neochmia temporalis* | 0 | 10.90 | 0.54 | Endocast |
| Passeridae | *Padda oryzivora* | 0 | 24.50 | 0.88 | Endocast |
| Passeridae | *Passer domesticus* | 0 | 28.49 | 0.92 | Endocast |
| Passeridae | *Passer griseus* | 1 | 23.90 | 0.97 | Published |
| Passeridae | *Passer hispaniolensis* | 4 | 27.13 | 0.93 | Endocast |
| Passeridae | *Passer montanus* | 0 | 21.38 | 0.79 | Published |
| Passeridae | *Passer rutilans* | 1 | 18.35 | 0.68 | Published |
| Passeridae | *Plocepasser mahali* | 0 | 43.30 | 1.27 | Published |
| Passeridae | *Ploceus cucullatus* | 0 | 40.90 | 1.42 | Endocast |
| Passeridae | *Poephila acuticauda* | 0 | 14.00 | 0.53 | Endocast |
| Passeridae | *Poephila cincta* | 0 | 16.10 | 0.49 | Published |
| Passeridae | *Poephila personata* | 0 | 11.80 | 0.52 | Endocast |
| Passeridae | *Prunella modularis* | 1 | 20.41 | 0.71 | Endocast |
| Passeridae | *Pyrenestes sanguineous* | 0 | 14.27 | 0.97 | Published |
| Passeridae | *Pytilia melba* | 1 | 13.50 | 0.59 | Published |
| Passeridae | *Pytilia phoenicoptera* | 2 | 14.50 | 0.37 | Published |
| Passeridae | *Spermophaga haematina* | 0 | 16.87 | 0.91 | Published |
| Passeridae | *Staganopleura guttata* | 1 | 19.00 | 0.65 | Published |
| Passeridae | *Taeniopygia bichenovii* | 1 | 10.50 | 0.41 | Endocast |
| Passeridae | *Taeniopygia guttata* | 0 | 12.00 | 0.46 | Endocast |
| Passeridae | *Uraeginthus bengalus* | 1 | 10.30 | 0.45 | Published |
| Passeridae | *Vidua paradisaea* | 1 | 22.20 | 0.66 | Endocast |
| Petroicidae | *Drymodes brunneopygia* | 0 | 38.70 | 0.98 | Endocast |
| Petroicidae | *Eopsaltria australis* | 0 | 19.60 | 0.89 | Endocast |
| Petroicidae | *Eopsaltria griseogularis* | 0 | 21.10 | 0.86 | Endocast |
| Petroicidae | *Melanodryas cucullata* | 0 | 24.30 | 0.83 | Endocast |
| Petroicidae | *Microeca fascinans* | 1 | 11.40 | 0.56 | Published |
| Petroicidae | *Microeca flavigaster* | 1 | 12.70 | 0.53 | Endocast |
| Petroicidae | *Pachycephalopsis poliosoma* | 0 | 38.30 | 1.10 | Endocast |
| Petroicidae | *Petroica goodenovii* | 1 | 8.70 | 0.38 | Endocast |
| Petroicidae | *Petroica multicolor* | 1 | 9.60 | 0.54 | Endocast |
| Petroicidae | *Tragellasia leucops* | 0 | 16.00 | 0.61 | Published |
| Pittidae | *Pitta brachyura* | 4 | 55.50 | 1.44 | Published |
| Pittidae | *Pitta erythrogaster* | 0 | 48.35 | 0.95 | Published |
| Pittidae | *Pitta guajana* | 0 | 69.51 | 1.42 | Published |
| Pittidae | *Pitta sordida* | 4 | 51.51 | 1.25 | Endocast |
| Pittidae | *Pitta versicolor* | 1 | 107.70 | 1.94 | Endocast |
| Pomatostomidae | *Pomatostomus halli* | 0 | 37.50 | 1.66 | Endocast |
| Pomatostomidae | *Pomatostomus isidorei* | 0 | 64.00 | 2.26 | Endocast |
| Pomatostomidae | *Pomatostomus ruficeps* | 1 | 56.00 | 2.43 | Published |
| Pomatostomidae | *Pomatostomus superciliosus* | 0 | 35.00 | 1.57 | Endocast |
| Pomatostomidae | *Pomatostomus temporalis* | 0 | 75.00 | 1.97 | Published |
| Ptilonorhynchidae | *Ailuroedus crassirostris* | 0 | 204.00 | 4.15 | Published |
| Ptilonorhynchidae | *Amblyornis macgregoriae* | 0 | 123.50 | 3.71 | Endocast |
| Ptilonorhynchidae | *Chlamydera nuchalis* | 1 | 199.50 | 5.19 | Endocast |
| Ptilonorhynchidae | *Ptilonorhynchus violaceus* | 1 | 217.00 | 4.89 | Endocast |
| Ptilonorhynchidae | *Sericulus chrysocephalus* | 0 | 100.50 | 3.46 | Endocast |
| Pycnonotidae | *Alophoixus pallidus* | 0 | 46.00 | 1.28 | Published |
| Pycnonotidae | *Chlorocichla flavicollis* | 0 | 45.30 | 1.53 | Published |
| Pycnonotidae | *Nicator chloris* | 0 | 38.20 | 1.33 | Published |
| Pycnonotidae | *Pycnonotus barbatus* | 0 | 35.90 | 1.17 | Published |
| Pycnonotidae | *Pycnonotus jocosus* | 0 | 27.40 | 0.96 | Published |
| Regulidae | *Regulus regulus* | 0 | 5.70 | 0.38 | Published |
| Rhinocryptidae | *Rhinocrypta lanceolata* | 0 | 63.60 | 1.45 | Published |
| Sittidae | *Sitta canadensis* | 0 | 10.50 | 0.59 | Endocast |
| Sittidae | *Sitta carolinensis* | 0 | 17.80 | 0.89 | Endocast |
| Sittidae | *Sitta europaea* | 0 | 23.03 | 1.11 | Published |
| Sittidae | *Sitta pygmaea* | 0 | 10.40 | 0.57 | Endocast |
| Sturnidae | *Acridotheres tristis* | 0 | 115.60 | 2.57 | Endocast |
| Sturnidae | *Aplonis metallica* | 1 | 61.00 | 1.64 | Published |
| Sturnidae | *Dumetella carolinensis* | 4 | 37.80 | 1.18 | Endocast |
| Sturnidae | *Gracula religiosa* | 0 | 192.00 | 3.81 | Published |
| Sturnidae | *Lamprotornis purpureus* | 0 | 115.50 | 2.20 | Published |
| Sturnidae | *Lamprotornis superbus* | 0 | 54.15 | 1.78 | Published |
| Sturnidae | *Margarops fuscatus* | 0 | 109.40 | 2.43 | Endocast |
| Sturnidae | *Margarops fuscus* | 0 | 70.80 | 1.60 | Endocast |
| Sturnidae | *Melanoptila glabirostris* | 0 | 35.00 | 1.24 | Endocast |
| Sturnidae | *Mimus polyglottos* | 2 | 45.20 | 1.43 | Endocast |
| Sturnidae | *Poeoptera lugubris* | 0 | 38.00 | 1.03 | Published |
| Sturnidae | *Sarcops calvus* | 0 | 142.00 | 2.90 | Endocast |
| Sturnidae | *Sturnus roseus* | 4 | 66.50 | 1.48 | Published |
| Sturnidae | *Sturnus vulgaris* | 3 | 109.40 | 2.24 | Endocast |
| Sturnidae | *Toxostoma curvirostre* | 0 | 78.40 | 2.21 | Endocast |
| Sylviidae | *Abroscopus albogularis* | 0 | 5.00 | 0.33 | Endocast |
| Sylviidae | *Acrocephalus arundinaceus* | 4 | 29.90 | 0.94 | Endocast |
| Sylviidae | *Acrocephalus melanopogon* | 2 | 11.27 | 0.41 | Published |
| Sylviidae | *Acrocephalus orientalis* | 4 | 23.98 | 0.87 | Published |
| Sylviidae | *Acrocephalus palustris* | 4 | 11.70 | 0.39 | Published |
| Sylviidae | *Acrocephalus schoenobaenus* | 4 | 10.80 | 0.47 | Endocast |
| Sylviidae | *Acrocephalus scirpaceus* | 4 | 12.30 | 0.48 | Published |
| Sylviidae | *Chaemaea fasciata* | 0 | 14.70 | 0.80 | Published |
| Sylviidae | *Cincloramphus cruralis* | 1 | 43.00 | 1.16 | Published |
| Sylviidae | *Cincloramphus mathewsi* | 1 | 25.00 | 0.93 | Published |
| Sylviidae | *Garrulax leucolophus* | 0 | 123.50 | 2.55 | Published |
| Sylviidae | *Heterophasia melanoleuca* | 0 | 32.60 | 1.20 | Published |
| Sylviidae | *Hippolais icterina* | 4 | 12.74 | 0.54 | Published |
| Sylviidae | *Illadopsis fulvescens* | 0 | 24.40 | 1.23 | Published |
| Sylviidae | *Leiothrix argentauris* | 0 | 26.20 | 0.97 | Published |
| Sylviidae | *Leiothrix lutea* | 0 | 21.80 | 0.95 | Published |
| Sylviidae | *Locustella fluviatilis* | 4 | 18.80 | 0.45 | Published |
| Sylviidae | *Locustella luscinioides* | 4 | 15.00 | 0.57 | Published |
| Sylviidae | *Macronous gularis* | 0 | 13.50 | 0.67 | Endocast |
| Sylviidae | *Megalurus palustris* | 0 | 33.30 | 1.18 | Endocast |
| Sylviidae | *Minla ignotincta* | 0 | 14.30 | 0.74 | Endocast |
| Sylviidae | *Phylloscopus bonelli* | 4 | 7.40 | 0.35 | Endocast |
| Sylviidae | *Phylloscopus collybita* | 4 | 8.02 | 0.38 | Published |
| Sylviidae | *Phylloscopus sibiliatrix* | 4 | 7.20 | 0.37 | Endocast |
| Sylviidae | *Phylloscopus trochilus* | 4 | 9.92 | 0.31 | Published |
| Sylviidae | *Pomatorhinus ruficollis* | 0 | 31.70 | 1.59 | Published |
| Sylviidae | *Pteruthius flaviscapis* | 0 | 39.00 | 1.55 | Published |
| Sylviidae | *Stachyris whiteheadi* | 0 | 19.10 | 0.85 | Endocast |
| Sylviidae | *Sylvia atricapilla* | 3 | 19.37 | 0.67 | Endocast |
| Sylviidae | *Sylvia borin* | 4 | 18.22 | 0.62 | Endocast |
| Sylviidae | *Sylvia communis* | 4 | 14.09 | 0.56 | Published |
| Sylviidae | *Sylvia curruca* | 4 | 12.82 | 0.53 | Published |
| Sylviidae | *Sylvia hortensis* | 4 | 22.25 | 0.79 | Published |
| Sylviidae | *Sylvia melanocephala* | 0 | 10.98 | 0.53 | Published |
| Sylviidae | *Sylvia nisoria* | 4 | 22.80 | 0.71 | Published |
| Sylviidae | *Yuhina diademata* | 0 | 12.00 | 0.80 | Published |
| Thamnophilidae | *Cercomacra tyrannina* | 0 | 16.60 | 0.77 | Published |
| Thamnophilidae | *Dystithamnus mentalis* | 0 | 11.20 | 0.73 | Endocast |
| Thamnophilidae | *Formicivora grisea* | 0 | 9.30 | 0.47 | Published |
| Thamnophilidae | *Gymnocichla nudiceps* | 0 | 32.80 | 1.10 | Published |
| Thamnophilidae | *Gymnopithys leucaspis* | 0 | 31.10 | 0.79 | Published |
| Thamnophilidae | *Hylophylax naevia* | 0 | 12.50 | 0.61 | Endocast |
| Thamnophilidae | *Hylophylax poecilonota* | 0 | 16.60 | 0.66 | Endocast |
| Thamnophilidae | *Hypocnemis cantator* | 0 | 10.00 | 0.65 | Endocast |
| Thamnophilidae | *Myrmotherula axillaris* | 0 | 7.40 | 0.40 | Endocast |
| Thamnophilidae | *Myrmotherula fulviventris* | 0 | 10.20 | 0.50 | Endocast |
| Thamnophilidae | *Phaenostictus mcleannani* | 0 | 51.10 | 1.11 | Published |
| Thamnophilidae | *Pithys albifrons* | 0 | 19.70 | 0.64 | Endocast |
| Thamnophilidae | *Pyriglena leuconota* | 0 | 32.80 | 0.96 | Endocast |
| Thamnophilidae | *Sakesphorus luctuosus* | 0 | 31.00 | 1.19 | Published |
| Thamnophilidae | *Taraba major* | 0 | 67.50 | 1.57 | Endocast |
| Thamnophilidae | *Thamnomanes caesius* | 0 | 14.20 | 0.63 | Endocast |
| Thamnophilidae | *Thamnophilus caerulescens* | 0 | 20.00 | 1.02 | Endocast |
| Thamnophilidae | *Thamnophilus punctatus* | 0 | 22.40 | 0.97 | Published |
| Tyrannidae | *Attila spadiceus* | 0 | 33.20 | 1.22 | Endocast |
| Tyrannidae | *Camptostoma obsoletum* | 0 | 8.00 | 0.37 | Published |
| Tyrannidae | *Chiroxiphia caudata* | 0 | 25.60 | 0.83 | Published |
| Tyrannidae | *Chiroxiphia linearis* | 0 | 18.50 | 0.70 | Endocast |
| Tyrannidae | *Cnemotriccus fuscatus* | 3 | 13.50 | 0.48 | Published |
| Tyrannidae | *Colonia colonus* | 1 | 16.80 | 0.49 | Endocast |
| Tyrannidae | *Contopus latirostris* | 0 | 10.60 | 0.39 | Published |
| Tyrannidae | *Contopus virens* | 4 | 14.20 | 0.47 | Published |
| Tyrannidae | *Elaenia frantzii* | 2 | 19.40 | 0.63 | Endocast |
| Tyrannidae | *Elaenia martinica* | 0 | 18.60 | 0.67 | Endocast |
| Tyrannidae | *Empidonax minimus* | 4 | 10.50 | 0.38 | Endocast |
| Tyrannidae | *Empidonax virescens* | 4 | 12.90 | 0.45 | Published |
| Tyrannidae | *Gymnoderus foetidus* | 0 | 275.00 | 3.66 | Published |
| Tyrannidae | *Hemitriccus margaritaceiventer* | 0 | 7.70 | 0.47 | Published |
| Tyrannidae | *Hymenops perspicillatus* | 3 | 23.10 | 0.79 | Endocast |
| Tyrannidae | *Legatus leucophaius* | 4 | 24.40 | 0.68 | Published |
| Tyrannidae | *Lessonia rufa* | 4 | 14.48 | 0.44 | Published |
| Tyrannidae | *Lipaugus vociferans* | 1 | 82.20 | 1.69 | Endocast |
| Tyrannidae | *Manacus candei* | 0 | 17.60 | 0.59 | Endocast |
| Tyrannidae | *Manacus manacus* | 0 | 19.00 | 0.61 | Endocast |
| Tyrannidae | *Manacus vitellinus* | 0 | 18.20 | 0.64 | Endocast |
| Tyrannidae | *Mecocerculus leucophrys* | 1 | 13.90 | 0.50 | Endocast |
| Tyrannidae | *Mionectes oligeneus* | 1 | 13.20 | 0.47 | Published |
| Tyrannidae | *Mitrephanes phaeocercus* | 1 | 8.60 | 0.33 | Endocast |
| Tyrannidae | *Muscisaxicola alpina* | 0 | 22.80 | 0.81 | Endocast |
| Tyrannidae | *Myiarchus cinerascens* | 4 | 28.80 | 0.80 | Endocast |
| Tyrannidae | *Myiarchus stolidus* | 0 | 19.30 | 0.67 | Endocast |
| Tyrannidae | *Myiarchus tuberculifer* | 3 | 18.70 | 0.68 | Endocast |
| Tyrannidae | *Myiarchus tyrannulus* | 3 | 35.30 | 1.02 | Endocast |
| Tyrannidae | *Myiobius barbatus* | 1 | 11.90 | 0.36 | Endocast |
| Tyrannidae | *Myiodynastes maculatus* | 1 | 45.90 | 1.17 | Endocast |
| Tyrannidae | *Myiopagis cotta* | 0 | 13.00 | 0.44 | Published |
| Tyrannidae | *Myiophobus fasciatus* | 3 | 9.90 | 0.38 | Endocast |
| Tyrannidae | *Myiozetetes similis* | 1 | 27.80 | 0.85 | Endocast |
| Tyrannidae | *Onychorhynchus coronatus* | 1 | 14.00 | 0.49 | Endocast |
| Tyrannidae | *Pachyramphus cinnamomeus* | 1 | 20.30 | 0.91 | Published |
| Tyrannidae | *Pachyramphus polychopterus* | 0 | 20.30 | 0.77 | Endocast |
| Tyrannidae | *Perissocephalus tricolor* | 0 | 339.50 | 4.87 | Endocast |
| Tyrannidae | *Phaeomyias murina* | 3 | 10.00 | 0.38 | Published |
| Tyrannidae | *Phytotoma rara* | 1 | 40.00 | 1.20 | Published |
| Tyrannidae | *Pipra erythrocephala* | 0 | 13.60 | 0.51 | Endocast |
| Tyrannidae | *Pipra fasciicauda* | 0 | 14.30 | 0.64 | Endocast |
| Tyrannidae | *Pipra mentalis* | 1 | 15.20 | 0.61 | Endocast |
| Tyrannidae | *Pitangus sulphuratus* | 3 | 70.20 | 1.32 | Endocast |
| Tyrannidae | *Platyrinchus concrominus* | 0 | 9.70 | 0.51 | Endocast |
| Tyrannidae | *Pseudocolopteryx flaviventris* | 2 | 7.40 | 0.35 | Endocast |
| Tyrannidae | *Pseudotriccus pelzelni* | 0 | 10.90 | 0.66 | Endocast |
| Tyrannidae | *Pyrocephalus rubinus* | 3 | 12.70 | 0.48 | Published |
| Tyrannidae | *Pyroderus scutatus* | 0 | 357.00 | 4.45 | Published |
| Tyrannidae | *Querula purpurata* | 0 | 101.80 | 2.21 | Endocast |
| Tyrannidae | *Rupicola peruviana* | 0 | 243.50 | 3.48 | Published |
| Tyrannidae | *Sayornis nigricans* | 2 | 18.60 | 0.51 | Published |
| Tyrannidae | *Sayornis phoebe* | 4 | 18.30 | 0.61 | Published |
| Tyrannidae | *Sayornis saya* | 4 | 21.70 | 0.63 | Endocast |
| Tyrannidae | *Schiffornis turdinus* | 0 | 30.80 | 1.11 | Endocast |
| Tyrannidae | *Tityra cayana* | 1 | 73.90 | 1.67 | Published |
| Tyrannidae | *Tityra semifasciata* | 0 | 79.30 | 1.95 | Endocast |
| Tyrannidae | *Todirostrum cinereum* | 0 | 6.40 | 0.32 | Endocast |
| Tyrannidae | *Tolmomyias sulphurescens* | 0 | 14.90 | 0.53 | Endocast |
| Tyrannidae | *Tyrannus savanna* | 4 | 28.60 | 0.76 | Published |
| Tyrannidae | *Tyrannus tyrannus* | 4 | 39.50 | 0.98 | Published |
| Tyrannidae | *Xolmis irupero* | 0 | 29.80 | 0.85 | Published |
| Tyrannidae | *Xolmis pyrope* | 1 | 42.80 | 1.33 | Endocast |
| Vireonidae | *Cyclarhis gujanensis* | 0 | 28.80 | 1.31 | Endocast |
| Vireonidae | *Vireo altiloquus* | 4 | 19.50 | 0.78 | Endocast |
| Vireonidae | *Vireo olivaceus* | 4 | 20.30 | 0.63 | Published |
| Vireonidae | *Vireo philadelphicus* | 4 | 12.20 | 0.48 | Published |
| Zosteropidae | *Zosterops japonicus* | 0 | 10.20 | 0.56 | Endocast |
| Zosteropidae | *Zosterops lateralis* | 1 | 10.6 | 0.49 | Endocast |
